# Severe destruction in vertebral osteomyelitis – risk factors and survival

**DOI:** 10.1007/s15010-025-02701-x

**Published:** 2025-11-24

**Authors:** Jan Philipp Hockmann, Nikolaus Kernich, Krishnan Sircar, Peer Eysel, Ada Hoffmann, Dorothee Jochimsen, Norma Jung, Ayla Yagdiran

**Affiliations:** 1 Department of Orthopaedic Surgery, Traumatology and Plastic-Reconstructive Surgery, University of Cologne, Faculty of Medicine and University Hospital Cologne, Joseph-Stelzmann-Str. 24, 50931 Cologne, Germany; 2 Institute for Medical Microbiology, Immunology and Hygiene (IMMIH), University of Cologne, Faculty of Medicine and University Hospital Cologne, Cologne, Germany; 3 Division of Infectious Diseases, Department I of Internal Medicine, University of Cologne, Faculty of Medicine and University Hospital Cologne, Cologne, Germany

**Keywords:** Vertebral osteomyelitis, Destruction, Pathogens, Risk factors, Survival, Obesity, Postoperative

## Abstract

**Purpose:**

The treatment of vertebral osteomyelitis (VO) is multifaceted. In most cases, patients are treated conservatively. In the past, it has already been shown that the quality of life is improved by surgery compared to conservative treatment. Recently it was also shown that surgical treatment of VO improves recurrence and mortality. Indications of a surgical approach can be relative or absolute. Absolute indications include, above all, severe bony destruction. The aim of this study is to identify risk factors for severe destruction in comparison to minor to or not to moderate destruction.

**Methods:**

This monocentric study included 355 patients with VO from the DWG- and the former European Spine Tango-Registry. The primary aim was to analyze the risk factors for severe destruction, the secondary aim was to determine the outcomes between the different degrees of severity.

**Results:**

The prediction model for severe destruction with the factors age ≥ 65 years, gender, CRP ≥ 10 mg/L, an ASA class above two, a BMI ≥ 25 kg/m^2^, a bacteremia, more than two comorbidities, more than one segment affected, detected pathogen, Staphylococcus aureus as pathogen, a previous injection or surgery was significant as a whole (Chi^2^(12) = 22.48, *p* = 0.032, *n* = 355). An ASA class of three and higher increased the risk of severe destruction by 1.77 times (*p* = 0.027), a BMI ≥ 25 kg/m^2^ reduced the risk by 0.6 times (*p* = 0.03) and a previous surgery reduced the risk by 0.5 times (*p* = 0.04). Survival was longer for mild to moderate destruction compared to severe destruction 142 (95%CI 125–142) days vs. 72 (95%CI 47–99) days (*p* = 0.001).

**Conclusions:**

Multimorbid patients with native VO exhibit more severe bony destruction. These patients are high-risk VO patients with an increased 1-year mortality rate. Therefore an early indication for surgical treatment may proof advantageous.

## Introduction

Vertebral osteomyelitis (VO) is an infection of the vertebral body in some cases with direct involvement of the intervertebral disc otherwise refered to as spondylodiscitis. Although not amongst the common sites for osteomyelitis, the demographic change leads to an increased incidence of VO due to the aging of the population [1]. The course of the disease is frequently severe with persistent impairments such as reduced Quality of life (QoL) [2–4]. The mortality is reported up to 20% within the first year [4, 5]. Depending on the demographic location and route of infection the pathogens responsible differ. In earlier times most often, granulomatous osteomyelitis occurred and still to date in some populations such as the study population of Gök et al. tuberculous and brucellar VO are common [6, 7]. In the western world pyogenic VO is most common with high prevalences of gram-positive pathogens such as *Staphylococcus aureus* [8–11]. These pathogens are also more common in the to date increased iatrogenous route of infection after spinal instrumentation. The diagnosis is complicated by initially mild symptoms [12]. Besides purulent infections with abscesses reaching neighboring structures such as the dura or for example the psoas muscle, VO is often associated with destruction of the vertebrae. The extend of the destruction can be classified based on Eysel and Peters [13].

Initially a deformation of the intervertebral space as a result of destruction or edema of the disc is present (Eysel and Peters 1). Subsequently, irregularities of the end plates occur due to progressive osteodestruction (Eysel and Peters 2). In later stages segmental kyphosis is present (Eysel and Peters 3) and at end stages extensive ankylosis and kyphosis can be observed (Eysel and Peters 4).

QoL of patients with VO can be improved by surgery compared to conservative treatment [3, 4]. Recently it was also shown that surgically treated VO improves recurrence and mortality [5, 14]. Indications of a surgical approach can be relative or absolute. Absolute indications include, above all, severe bony destruction [3]. There is no study to date evaluating the risk factors for severe destruction.

The aim of this study is to identify risk factors for severe destruction in VO patients to enable clinicians to identify patients who are in the necessity of surgery earlier to improve the outcome. In addition the survival between different extends of destruction was evaluated.

## Methods

This study was conducted in accordance with the Declaration of Helsinki and was approved by the institutional review board (IRB) of the study institution with the number 09-182.

The “Deutsche Wirbelsäulengesellschaft” (DWG) and the former European Spine Tango-Registry of the study location was retrospectively screened for patients with VO. Between March 2008 and January 2020, in total 355 patients were included. Only patients older than 18 years with a full dataset were included.

For evaluation of the effect of severity two groups of destruction were formed. Severe destruction (SD) including Eysel types three and above as well as mild to moderate destruction (MD) comprising Eysel types zero, one and two.

For the evaluation age, sex, body mass index (BMI), length of hospital stay, affected spinal segment, number of affected segments, American Society of Anesthesiologists Physical Status Classification System (ASA), positive blood cultures, causative pathogens, preoperative hemoglobin (Hb), C-reactive protein (CRP) and leukocyte levels, Charlson comorbidity index (CCI), presence of psoas abscess or empyema, preceding intervention or surgery, neurological deficit and comorbidities were evaluated.

Variables were selected for multivariate analysis based on clinical relevance and known from to be associated with the outcome of VO. Groups were formed for the regression analysis [10]. ASA was dichotomized as ≤ 2 vs. >2, since an ASA score greater than 2 indicates the presence of at least one severe systemic disease. Age was categorized as < 65 vs. ≥65 years, as 65 years is a widely used threshold to distinguish older adults in clinical research [15, 16]. The number of affected segments was categorized as one vs. more than one to differentiate localized from more extensive disease involvement. CRP was dichotomized at 10 mg/L, which is a commonly accepted cut-off to indicate clinically relevant systemic inflammation. BMI was categorized as < 25 vs. ≥25 kg/m², according to the World Health Organization definition of overweight [17].

Statistical analysis was performed with IBM SPSS Statistics Version 29.0 for Mac (IBM Corp, Armonk, NY). Graphics were done with GraphPad Prism 9.5.1. Normal distribution was evaluated by Kolmogorov-Smirnov and Shapiro-Wilk. We report average ± standard deviation or median (minimum–maximum). Follow-up was performed on scheduled follow-up visits or a phone call. Survival was analyzed by Kaplan-Meier curves, censoring was made on the day of death.

Mann-Whitney-U-test was performed to identify differences between groups and for proportions Chi-Square or Fisher´s exact test were calculated. Binary-logarithmic regression was performed for analysis of risk factors. A value of *p* < 0.05 was considered to be statistically significant.

## Results

Cohort characteristics are depicted in Table 1.

**Table 1:** Cohort characteristics

Characteristics	Value
Age, *mean ± standard deviation years*	67.23 ± 12.09
Sex, *n (%)*	
male	232 (65.35)
female	123 (34.65)
BMI, *mean ± standard deviation* kg/m^2^	26.84 ± 6.12
Hb at admission, *mean ± standard deviation g/dl*	11.24 ± 2
CRP at admission, *mean ± standard deviation mg/L*	94.79 ± 90.91
Leucocytes at admission, *mean ± standard deviation ×10*^*9*^*/L*	10.14 ± 5.56
Treatment, *n (%)*	
surgery	310 (87.32)
conservative	45 (12.68)
CCI, *n* (%)	
< 2	108 (69.58)
≥ 2	247 (30.42)
Positive blood cultures, *n* (%)	
Yes	128 (36.06)
No	226 (63.66)
Neurological deficit, *n* (%)	
Yes	81 (22.8)
No	274 (77.18)
Psoas abscess, *n* (%)	
Yes	79 (22.3)
No	276 (77.8)
Empyema., *n* (%)	
Yes	118 (33.2)
No	237 (66.8)
Affected segments, *n* (%)	
< 2	278 (78.3)
≥ 2	77 (21.7)
Prior infiltration of the spine, *n* (%)	
Yes	43 (12.1)
No	312 (87.9)
Prior spine surgery, *n* (%)	
Yes	71 (20)
No	284 (80)
Patient conditions, *n* (%)	
Diabetes mellitus	84 (23.7)
Oncological disease / Cancer diagnosis	71 (20)
Immunosuppression	27 (7.6)
Chronic obstructive pulmonary disease	35 (9.9)
Rheumatic disease	23 (6.5)
Heart failure	57 (16.1)
Chronic kidney disease	54 (15.2)
Osteoporosis	13 (3.7)
Causative pathogen, *n* (%)	
S. aureus (MSSA)	95 (26.8)
- MRSA	11 (3.1)
None detected	92 (25.9)
CoNS	53 (14.9)
Gram negative pathogens	25 (7.0)
Enterococcus faecalis	20 (5.6)
Streptococci spp.	19 (5.4)
Propionibacterium spp.	11 (3.1)
Mycobacterium spp.	9 (2.5)
Anaerobic pathogens	8 (2.3)
Candida spp.	3 (0.8)
Mixed	3 (0.8)
Brucella spp.	3 (0.8)
Pseudomonas aeruginosa	2 (0.6)
Corynebacteria spp.	1 (0.3)

CCI = *Charlson comorbidity index;* CoNS = Coagulase-Negative Staphylococci; MRSA = Methicilin-resistant S. aureus; BMI = Body Mass Index; Mycobacterium spp. = Non-tuberculosis and tuberculosis complex mycobacteria

Most of the patients showed signs of mild to moderate destruction 63.1% (*n* = 224). There was no significant difference in age between both groups SD 70 (20–92) years vs. MD 68 (26–91) years (*p* = 0.15). The laboratory findings preoperatively showed no significant differences. The CRP was 67.1 (3.4–412.4) mg/l in the SD group and 63 (2–471.1) mg/l in the MD group (*p* = 0.32). Leukocytes were 9.09 (2.39–23)10^9^/L in the SD group vs. 9.13 (2.91–78.63)10^9^/L in the MD group (*p* = 0.76). No significant difference was observed for preoperative Hb-levels SD 10.9(6.7–16) g/dl vs. MD 11.2(5.5–16.4) g/dl (*p* = 0.07). A significant difference between both groups was observed for the BMI (*p* = 0.001). It was higher in the MD group 26.8 (16–54.6) kg/m^2^ vs. the SD group 24.45 (15.4–48.9) kg/m^2^. Comorbidities showed no significant difference between both groups SD 1 (0–4) vs. MD 1(0–7) (*p* = 0.06). Pre-existing medical conditions were also evaluated. There was no statistically significant difference between both groups regarding diabetes mellitus (*p* = 0.61), oncologic disease (*p* = 0.06), immunosuppression (*p* = 0.21), chronic obstructive pulmonary disease (*p* = 0.69), rheumatic diseases (*p* = 0.5), heart failure (*p* = 0.99), chronic kidney disease (= 0.06), or osteoporosis (*p* = 0.73). The CCI was significantly higher in the SD group 4 (0–11) vs. 3 (0–12) (*p* = 0.001). No significant difference was shown for the affected segments SD 1(1–6) vs. MD 1(1–5) (*p* = 0.97). Significantly more patients underwent previous surgery in the MD group 23.66%(*n* = 53) vs. 13.74% (*n* = 18) (*p* = 0.02). There was no significant difference in the prevalence of previous injections (*p* = 0.19).

The prediction model for severe destruction with the factors age ≥ 65 years, gender (male), CRP ≥ 10 mg/L, an ASA class above two, a BMI ≥ 25 kg/m^2^, bacteremia, more than two comorbidities, more than one segment affected, detected pathogen, *Staphylococcus aureus* as pathogen, a previous injection or surgery was significant as a whole (Chi^2^(12) = 22.48, *p* = 0.032, *n* = 355). Model performance indicated modest explanatory power (Nagelkerke R² = 0.08). No relevant multicollinearity was detected, as all variance inflation factors (VIFs) ranged from 1.02 to 1.17.

**Fig. 1:**
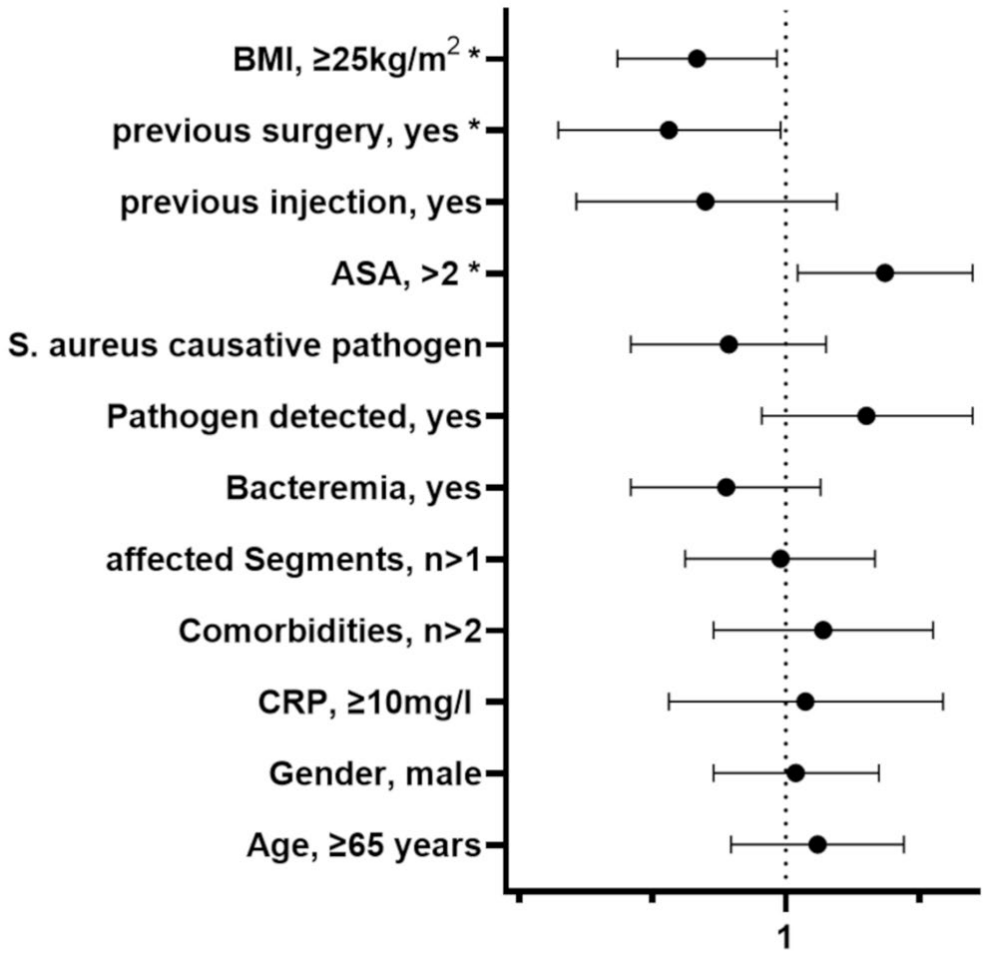
Odds-Ratios of the regression analysis. (BMI = Body Mass Index; American Association of Anesthesiologists risk classification, CRP = C-reactive protein)

There were three parameters significantly associated with severe destruction. An ASA score of over two (OR 1.77, 95% CI 1.07–2.93), previous surgery (OR 0.51, 95% CI 0.27–0.97) and a BMI of over 25 kg/m² (OR 0.60, 95% CI 0.38–0.95). No significant associations were observed for age over 65 years (OR 1.20, 95% CI 0.73–1.97), male gender (OR 1.06, 95% CI 0.66–1.71), CRP over 10 mg/L (OR 1.12, 95% CI 0.51–2.47), more than two comorbidities (OR 1.24, 95% CI 0.66–2.33), more than one affected spinal segment (OR 0.97, 95% CI 0.56–1.67), bacteremia (OR 0.71, 95% CI 0.41–1.22), a detected pathogen (OR 1.59, 95% CI 0.87–2.93) or *Staphylococcus aureus* as the causative pathogen (OR 0.72, 95% CI 0.41–1.26). The Odds-ratios are shown in Fig. 1.

In the SD group 28.24% (*n* = 37) patients had a neurological deficit, 19.85% (*n* = 26) had an abscess and 28.24% (*n* = 37) had an empyema. In the MD group 19.64% (*n* = 44) had a neurological deficit, 23.66% (*n* = 53) had an abscess and 36.16% (*n* = 81) had an empyema. None of these complications differed significantly between both groups (*p* = 0.06, *p* = 0.4, *p* = 0.13). Significantly less patients were treated operatively in the MD group 83.9% (*n* = 188) then in the SD group 91.6% (*n* = 120) (*p* = 0.03). The hospitalization was significantly longer for patients with severe destruction 32 (7–138) days compared to 26.5 (3–193) days (*p* = 0.001).

**Fig. 2:**
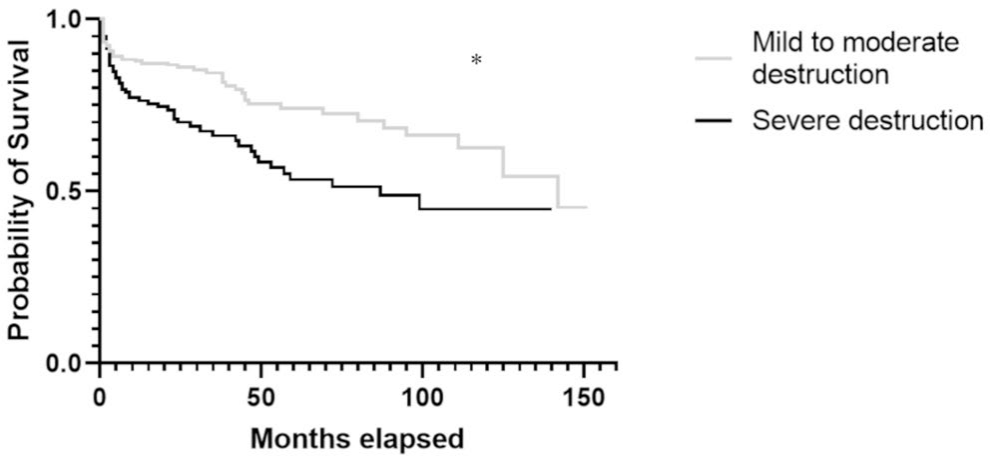
Kaplan-Meier curve for the survival of both groups

The median follow-up was 43.1 (0–4617) days. The mortality after one year was 14.5% (*n* = 29) in the MD group and 26.23% (*n* = 32) in the SD group with a significant difference between both groups (*p* = 0.01). Survival of both groups is shown in Fig. 2.

The Log-Rank test revealed a significant difference of the survival between both groups (*p* = 0.001). The median survival was 142 (95%CI 125–142) months in the MD group and 72 (95%CI 47–99) months in the SD group.

## Discussion

This study is the first study to evaluate risk factors for severe destruction in severe VO. The presented data demonstrates the characteristics of patients with different extends of destruction. Patients showed signs of infectious disease states with elevated CRP levels on average although within a great range and no difference between both groups. As the leukocyte levels were also normal it emphasizes the often mild initial process of this infectious disease. Though patients might clinically present with extensive infectious processes resembled by up to one third of patients in this cohort with abscesses, empyema’s or neurological deficit. These findings coincide with the studies present [8, 18, 19, 6, 11, 10]. The presented data also supports the more frequent occurrence in middle aged men. Although advancements of microbiological evaluation lead to improved pathogen detection, the absence of a positive finding is still a problem and for example calculative antibiotic therapy has to be administered frequently [11, 8]. During this studies period the chosen empiric antibiotic therapy was ceftriaxone and flucloxacillin although in the meantime the most common and local regime changed to ampicillin and sulbactam.

Previous studies already demonstrated that there is no significant difference of the level of destruction between patients with or without previous injection or a detected pathogen [20, 3].

The cut-off for the BMI was chosen based on the World Health Organizations (WHO) classification for overweight. Not only was the BMI significantly different in both groups but it also proved to be a significant factor in predicting severe destruction. This finding should be interpreted cautiously, as the underlying mechanisms remain unclear and the analysis is hypothesis-generating. One possible explanation might be that overweight patients experience earlier symptoms even in mild or moderate destruction due to increased loading of the affected spinal segments [21]. Schoof et al. were able to show that in cases of spondylodiscitis, obesity leads to more severe courses which could also lead to a diagnosis in an earlier state of destruction [22]. These interpretations are speculative and require confirmation in future studies. The reduced risk of a severe destruction after a previous surgery could be a result of earlier patient-doctor contact due to follow-up visits and or the earlier presentation with pain due to pre-existing defects. Although previous studies were able to show no difference or a tendency for a longer time until diagnosis from symptom onset for patients with previous surgeries especially in spondylodiscitis [23, 9]. It could also be a limitation of the classification system used, as kyphosis could be more difficult to detect after spondylodesis or other stiffening operations leading to an underestimated destruction in patients with previous stabilizing surgeries in the affected segments. The effect of the ASA classification as a risk factor for severe destruction corresponds with the reduced survival and longer hospitalization of these patients. Those patients seem to have a significant burden on their health condition based on the destructive process of VO and not only on preexisting conditions. There was a significant difference for the CCI but not for the number of comorbidities nor were more than two comorbidities significantly influential on the risk of severe destruction.

Although survival overall and one-year survival were significantly lower for patients with severe destruction, Yagdiran et al. were able to show that the degree of destruction had no significant influence on the return to work [2]. *S. aureus* was the most common causative pathogen identified from microbiological culture. It is not only one of the most common pathogens in osteomyelitis but was frequently found in other studies of VO [24–26]. The rate of determination of the causative pathogen remains low and a broad spectrum of detected pathogens may lead to the insignificance as a risk factor of *S. aureus*.

Limitations of this study encompass the retrospective, monocentric study design. Patients were mostly referred for operation and treated operatively. Mild forms of destruction might be underrepresented as they were not referred to the study center. Although there was still an asymmetry of the group size towards MD. Another limitation is that the grouping of patients for regression analysis involves the risk of bias. Cut-off values of pre-existing studies were used to reduce this risk. Multicentered studies involving also basic care teams should confirm the presented findings.

Overall, the presented data supports the previous findings of patient’s characteristics with VO. The reduced outcome of patients with severe destruction emphasizes the need for early diagnosis and treatment initiation.

## Conclusion

Multimorbid patients with native VO exhibit more severe bony destruction. These patients are high-risk VO patients with an increased 1-year mortality rate. Therefore an early indication for surgical treatment may proof advantageous.

## Data Availability

The data that support the findings of this study are not available due to restrictions on privacy and ethical restrictions.
